# Neurological side effects after SARS-CoV-2 vaccinations require thorough and comprehensive investigations

**DOI:** 10.3325/cmj.2024.65.161

**Published:** 2024-04

**Authors:** Josef Finsterer

**Affiliations:** Neurology & Neurophysiology Center, Vienna, Austria

*fifigs1@yahoo.de*

To the Editor,

We read with interest the article by Salai et al about an email survey of 10 patients with muscle twitches or migraine auras after SARS-CoV2 vaccination (SC2V) (1). Patients who developed fasciculations (n = 10) or aura without migraine (n = 5) completed a structured questionnaire about their symptoms, treatment, and disease progression an average of 321 days after the onset of neurological symptoms (1). Half of the patients developed migraine auras without headache, and all patients developed fasciculations (1). The study is impressive, but some points require further discussion.

The first point is that the included patients were not examined by a neurologist. To assess whether these patients actually had fasciculations or rather myoclonus, dystonia, tremor, ataxia, or even focal seizures, they would have to be referred for a neurological examination. Since neurological side effects, including those reported by the included patients, are common after SC2V (2), and laypeople cannot differentiate between these neurological side effects, it is inevitable that they should be evaluated by a specialist to rule out central nervous system or peripheral nervous system causes of the symptoms.

The second point is that none of the ten patients were evaluated by instrumental examinations such as cerebral imaging, electroencephalography (EEG), or cerebrospinal fluid (CSF) studies. To rule out stroke, bleeding, demyelinating disorder, or encephalitis, cerebral magnetic resonance imaging or magnetic resonance angiography is mandatory. To rule out venous sinus thrombosis, magnetic resonance venography is mandatory. To rule out encephalitis or demyelinating disease, it is imperative to test the CSF for pleocytosis, protein, lactate, blood brain barrier permeability, cytokines, chemokines, glial markers, or even antibodies associated with autoimmune encephalitis. To rule out seizures, it is imperative to record an EEG to determine whether twitches ultimately correlate with epileptiform discharges.

The third point is that the latency of 4-36.5 days between SC2V and onset of neurological impairment was quite long, at least in some patients. With a latency of 36.5 days one should consider that causes other than SC2V could be responsible for the neurological abnormalities. Were all included patients subjected to a thorough examination immediately after onset of neurological impairment? The authors report that one patient attributed neurological symptoms not to SC2V, but to a SARS-CoV-2 infection he suffered after SC2V. This particular patient should have been excluded from the evaluation.

The fourth point is that for the five patients with aura it was not stated what type of aura they suffered from. Was it visual (eg, retinal migraine) or sensory aura, dysarthria or aphasia, brainstem symptoms, or motor aura (eg, hemiplegia)? Since aura can also be a manifestation of epilepsy, it is imperative to thoroughly rule out seizure activity as a cause of these manifestations. Were EEG recordings performed on any of the five patients? Was the history of epilepsy positive in any of the five patients?

The fifth point is that 11 patients are mentioned in Table 1, while only 10 patients were mentioned in the result section. This discrepancy should be resolved.

The sixth point is that the latency between the appearance of neurological symptoms and the survey was, on average, almost one year. The disadvantage of such a long latency is that some patients may not have been able to accurately remember previous symptoms. Was the description of the symptoms confirmed by additional history taking from relatives or close friends?

In summary, the interesting study has limitations that put the results and their interpretation into perspective. Clarifying these weaknesses would strengthen the conclusions and could improve the study. Before attributing fasciculations and aura to SC2V, neurological, clinical, and instrumental examinations are necessary to assess the nature of the twitches and thoroughly rule out all possible differential causes in addition to SC2V.
